# Correlation of exhaled breath temperature with bronchial blood flow in asthma

**DOI:** 10.1186/1465-9921-6-15

**Published:** 2005-02-10

**Authors:** Paolo Paredi, Sergei A Kharitonov, Peter J Barnes

**Affiliations:** 1 Department of Thoracic Medicine, National Heart and Lung Institute, Faculty of Medicine, Imperial College, London, UK

**Keywords:** asthma, nitric oxide, temperature, bronchial blood flow, inflammation.

## Abstract

In asthma elevated rates of exhaled breath temperature changes (Δe°T) and bronchial blood flow (Q_aw_) may be due to increased vascularity of the airway mucosa as a result of inflammation.

We investigated the relationship of Δe°T with Q_aw _and airway inflammation as assessed by exhaled nitric oxide (NO). We also studied the anti-inflammatory and vasoactive effects of inhaled corticosteroid and β_2_-agonist.

Δe°T was confirmed to be elevated (7.27 ± 0.6 Δ°C/s) in 19 asthmatic subjects (mean age ± SEM, 40 ± 6 yr; 6 male, FEV_1 _74 ± 6 % predicted) compared to 16 normal volunteers (4.23 ± 0.41 Δ°C/s, p < 0.01) (30 ± 2 yr) and was significantly increased after salbutamol inhalation in normal subjects (7.8 ± 0.6 Δ°C/ s, p < 0.05) but not in asthmatic patients. Q_aw_, measured using an acetylene dilution method was also elevated in patients with asthma compared to normal subjects (49.47 ± 2.06 and 31.56 ± 1.6 μl/ml/min p < 0.01) and correlated with exhaled NO (r = 0.57, p < 0.05) and Δe°T (r = 0.525, p < 0.05). In asthma patients, Q_aw _was reduced 30 minutes after the inhalation of budesonide 400 μg (21.0 ± 2.3 μl/ml/min, p < 0.05) but was not affected by salbutamol.

Δe°T correlates with Q_aw _and exhaled NO in asthmatic patients and therefore may reflect airway inflammation, as confirmed by the rapid response to steroids.

## 

Asthma is an inflammatory disease of the airways. Vasodilatation is a critical feature of inflammation, and angiogenesis and vascular remodelling are features of chronic inflammatory diseases, such as asthma [1]. The increased vascularity of the airways in asthma [2] is partly due to the elevated number of vessels associated with angiogenesis and partly due to vasodilation caused by the release of vasodilator mediators, such as, histamine, bradykinin [3], and nitric oxide (NO) [4]. In a recent study we have found that patients with asthma have higher increases of exhaled breath temperature (Δe°T) compared with normal subjects and that this is correlated to the concentration of exhaled nitric oxide (NO) [5]. Therefore, we suggested that patients with asthma have high Δe°T. and that this may be due to airway inflammation and elevated levels of NO. In the present study we hypothesise that elevated Δe°T may result from increased heat exchange in the airways due to elevated bronchial blood flow (Q_aw_) caused by inflammation and airway remodelling. To test this, we investigate the relationship between airway inflammation as assessed by exhaled NO, with Q_aw _and Δe°T measured non-invasively. Q_aw _is an expression of bronchial blood flow, whereas Δe°T reflects the rate of temperature increase in the exhaled breath. We hypothesised that Q_aw _changes may contribute to the levels of Δe°T and we studied their relationship considering that, potentially, minor changes of bronchial blood flow may not affect exhaled breath temperature.

Elevated levels of exhaled NO in asthma [6,7] are likely to be due to the activation of the inducible form of NO synthase (iNOS) by inflammatory cytokines [8] and therefore, reflect airway inflammation. Because NO also regulates bronchial vascular tone [9] and may increase bronchial blood flow [10,11] we measured its concentration in the exhaled breath as a marker of inflammation and we analysed its relationship with Q_aw _and Δe°T.

The measurement of Q_aw _is non-invasive and was standardised and adapted from a previously validated technique [12,13]. Δe°T and bronchial blood flow (Q_aw_) were also measured non-invasively allowing us to make repeated measurements and to study the interactions of these two markers and exhaled NO.

The inhalation of corticosteroids has been shown to have an acute vasocostrictive effect on the bronchial circulation [14] and the measurement of Q_aw _has been advocated to assess airway steroid sensitivity. In order to evaluate further the correlation of Q_aw _and Δe°T with inflammation, we studied the acute effect of inhaled budesonide on these parameters and their reciprocal changes. Furthermore, to validate our methods, we also evaluated the vasodilating effect of the short-acting β_2_-agonist salbutamol.

## Methods

### Patients

Nineteen asthmatic patients were studied (7 male, age 40 ± 5 yr, FEV_1 _74 ± 6 %, 9 patients were on inhaled steroid treatment and 10 patients had mild persistent asthma and were on β_2_-agonist inhalers only). These two groups of patients were chosen because they are representative of the larger majority of asthmatic patients; in addition this allowed us to verify cross sectionally the effect of inhaled steroids on bronchial blood flow and exhaled NO.

We also examined 16 control subjects (age 30 ± 2 yr, 8 male) recruited from our outpatient clinic and from volunteers (Table 1). Most of these subjects had previously taken part in other published studies. The diagnosis of asthma was established in each patient according to American Thoracic Society criteria [15]. Patients with acute chest infection or disease exacerbation during the month before enrolment were excluded. Patients with history of diabetes, liver disease, heart failure, lung cancer, or alcohol/drug abuse were not eligible for the study. All subjects were life-long non-smokers. All asthmatic patients refrained from using β_2 _agonists and corticosteroids for at least 12 h prior to the study. The tests were carried out at ambient temperature between 23 and 25C° and humidity 60% and 65%.

**Table 1:** PATIENT CHARACTERISTICS

	Asthma not steroid treated (n = 10)	Steroid treated asthma (n = 9)	Normals (n = 16)
Age (years)	37 ± 7	45 ± 7	30 ± 2
Sex (M/F)	4/6	3/6	8/8
FEV_1 _(% predicted)	93 ± 10	75 ± 11	97 ± 9
Smokers	0	0	0
Ex smokers	0	0	0
Therapy:			
Inhaled β-drenergics	10	10	0
Theophylline	0	0	0
Inhaled steroids	0	10	0
Oral steroids	0	0	0

*Definition of abbreviations:*; FEV_1 _= forced expiratory volume in one second. Values are means ± SEM.

### Study design

The study was approved by the Brompton and Harefield NHS trust Ethics Committee. After a clinical examination was carried out, Δe°T, Q_aw _and exhaled NO were measured at least after one hour of rest in the laboratory. This was followed by spirometry.

Eight asthmatic subjects and eight normal volunteers agreed to have Δe°T and Q_aw _measured after the inhalation of budesonide 400 μg and salbutamol 200 μg or placebo, which were administered in three different visits. The measurements were repeated every 15 minutes for 1 h after budesonide and placebo inhalation and at baseline and every 10 minutes for half an hour after the inhalation of salbutamol.

### Exhaled breath temperature measurement

During a flow and pressure controlled single breath exhalation exhaled breath temperature gradients were measured as previously described [5]. As previously shown [5], during a flow and pressure controlled exhalation from total lung capacity through a 2.77 mm mouthpiece[16] exhaled breath temperature was measured by a fast response (1 ms) high accuracy (0.015 ± 0.027°C), thermometer (Picotech Ltd) interfaced with a computer by a single channel Picotech Oscilloscope (model ADC 42, resolution 12 bits) allowing online recording of exhaled breath temperature.

In a preliminary study exhaled breath temperature tracings were analyzed mathematically. The tracings proved to have an exponential rise and the point at 63% of the total temperature increase was chosen to study the slope of the curves because it represents two time constants of the maximal °T change and therefore allows a better mathematical characterization of the tracings before plateau.

The time constant of the thermometer response was found by measuring the time for the temperature to reach 63% of the final reading. This avoided large errors in estimating when the asymptotic final reading had been reached.

The exhaled breath temperature changes exponentially with time. The shape of the curve depends upon the *time constant *(T). In one time constant the response reaches 63% of its final change.

The response is of the form



Where *V*_2 _is the final value, *V*_1 _the initial value, and t is the time after exhalation was started. As t > infinity, the exponential term > 0. Hence the response rises asymptotically to its final value. When performing experiments to determine the time response of a system, it is difficult to tell at which time the final value is obtained, since there will be a small change in the response for a relatively long time as the asymptote is approached. Jitter and noise in the signal will also add to this problem. It is easier to estimate the asymptotic value and find the time at which a certain percentage of this is reached. Various percentages are possible, the most commonly used in physics and biology is the *time constant*, although in some applications (particularly electronic engineering) the *rise time *to 95% of the final value is used.

The 63% arises because when t = T, the expression above becomes

(*V*_2_-*V*_1_)(1-1/e) = (*V*_2_-*V*_1_)(1-0.37) = 0.63(*V*_2_-*V*_1_)

In n time constants the percentage reached is 100 × (1-1/e^n^)

which, approaches 98% after about 5 time constants.

The rate of temperature increase (Δe°T) calculated between the beginning of exhalation and 63 % of the total temperature increase (a/b, where "*a*" is 63% of Δ°T and "*b*" the time to reach "*a*") proved to be the more reproducible parameter to characterize the curves.

We evaluated the effect of different exhalation flow rates, distance of the thermocouple from the edge of the mouthpiece and ambient temperature on Δe°T and end-expiratory plateau temperatures. We found that Δe°T but not plateau temperatures were elevated at low (2–3 L/min) compared to higher (5–6 L/min) exhalation flow rates (6.25 ± 0.4°C/s and 4.45 ± 0.8°C/s, p < 0.01) in 8 normal subjects. When the thermocouple was inserted close to the edge of the mouthpiece (1 cm) the Δe°T was significantly higher (7.15 ± 0.2°C/s) compared to when it was located farther (2 cm) (4.45 ± 0.8°C/s, p < 0.01). There was a tendency for faster Δe°T when the subjects were starting exhaling from higher baseline ambient temperatures but this was not significant for temperature changes within ± 3°C (5.05 ± 0.8°C/s at 28°C and 4.45 ± 0.8°C/s at 22°C p > 0.05). The volume ventilation did not influence the Δe°T value. The difference in exhaled breath Δe°T and plateau temperatures measured during two successive collections at five minutes intervals (single session variability) was 4.4%, while between sessions variability (n = 6, one day interval) was 6.8%. The reproducibility of the test was confirmed by the Bland and Altman test [17].

### Exhaled NO measurements

Exhaled NO was measured using a modified chemiluminescence analyzer (model LR2000; Logan Research, Rochester, Kent) as previously described [18].

### Bronchial blood flow

**Figure 1:**
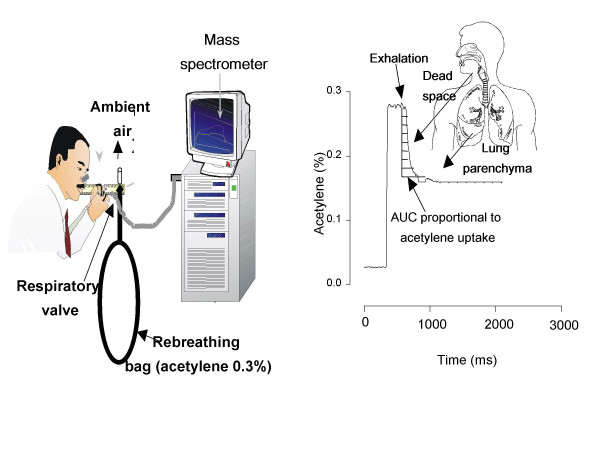
Method for the measurement of bronchial blood flow (Q_aw_). Subjects inhale 60% of their vital capacity from a reservoir containing acetylene 0.3% and then exhale into a mass spectrometer (Panel A). Panel B shows a tracing of acetylene, the area under the curve, corresponding to the conducting airways, is proportional to airway blood flow.

We modified a previously validated soluble inert gas uptake method to measure Q_aw_, using acetylene rather than the potentially explosive dimethylether [12,13]. The subjects were sitting in front of a valve system inhaling through a mouthpiece (with nose clips on) initially room air and then a gas mixture from a Teflon bag containing 35% O2, 0,3% acetylene, 5% sulphur hexafluoride, CO 3% and a balance of nitrogen. During the exhalation the concentration of acetylene was measured directly online by a mass spectrometer, and Q_aw _was calculated from the Fick principle (dilution of acetylene concentration) (Figure 1), the area under the curve (AUC) being inversely proportional to the bronchial blood flow. Q_aw _was expressed as μl/ml/min representing the volume of blood per volume of dead space per time.

Previous studies have used dimethyl ether instead of acetylene for the measurement of bronchial blood flow. In the current study we used acetylene because of the higher explosive potential of dimethylether when in contact with O_2_. This method was previously validated in the sheep where the invasive inoculation of radioactive micro spheres directly into the bronchial circulation provided similar results [19]. The use of micro spheres is considered the Gold Standard for the measurement of blood flow, however, this method is invasive and presents limitations such as the recirculation of the radioactive spheres.

Acetylene and diethyl ether present similar blood solubility and affinity for haemoglobin when measured at the same temperature [20]. Gas exchange efficiency is largely dependent on solubility. Because these two gases have similar physicochemical characteristics we assume that they can be used interchangeably. This is further confirmed by the finding of a similar range of bronchial blood flow and similar response to corticosteroids (vasoconstriction) and beta 2 agonists (vasodilatation) in this study compared to previously published studies which used the dimethyl ether method.

### Statistics

Comparisons between groups were made by one-way analysis of variance (ANOVA).. Data were expressed as means ± standard error of mean. The relationship between the exhaled breath temperature, Q_aw, _NO and FEV_1 _were tested with the linear correlation coefficient.

## Results

### Bronchial blood flow (Q_aw_)

**Figure 2:**
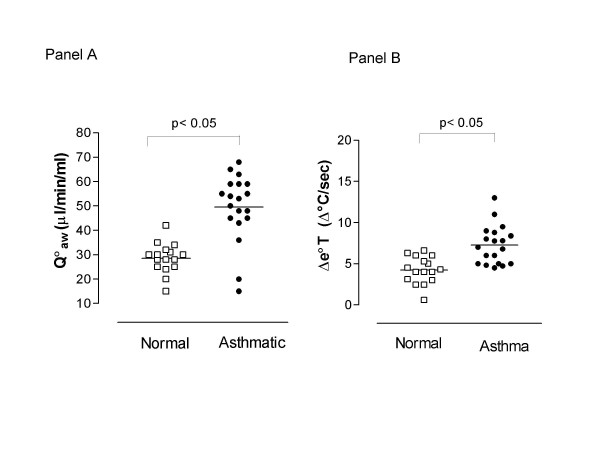
Levels of bronchial blood flow (Q_aw_) (Panel A) and exhaled breath temperature gradients (Δe°T) (Panel B) in normal subjects (□) and patients with asthma (•).

**Figure 3:**
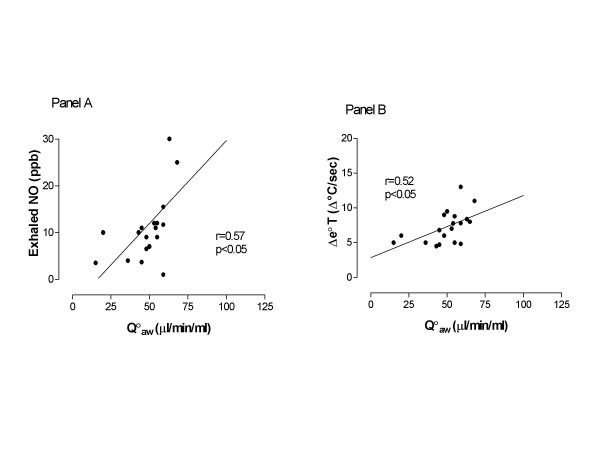
Correlation of bronchial blood flow (Q_aw_) with exhaled nitric oxide (NO) (Panel A) and exhaled breath temperature gradients (Δe°T) (Panel B) in patients with asthma.

Q_aw _was elevated to a similar extent in patients with mild persistent and moderate asthma (on regular inhaled corticosteroids and β_2_-agonists) (46.0 ± 51 μl/ml/min) and patients with mild intermittent asthma (on β_2_-agonists as needed only) (52.64 ± 3.0 μl/ml/min, p > 0.05) compared to normal subjects (31.56 ± 1.6 μl/ml/min, p < 0.01, Figure 2, Panel A). Q_aw _was correlated with exhaled NO (r = 0.57, p < 0.05, Figure 3, Panel A) and Δe°T (r = 0.52, p < 0.05, Figure 3, Panel B).

### Exhaled air temperature

Δe°T was higher in asthmatic patients (7.27 ± 0.6 Δ°C/ sec) compared to normal subjects (4.23 ± 0.41 Δ°C/ sec, p < 0.01, Figure 2, Panel B) and was not statistically different in steroid treated (7.56 ± 0.99 Δ°C/ sec) compared to untreated patients (6.83 ± 0.78 Δ°C/ sec, p > 0.05).

### Exhaled NO

NO levels were elevated in asthmatic subjects not on steroid treatment (15.6 ± 2.8 ppb) compared to steroid treated patients (7.5.6 ± 2.3 ppb, p < 0.05) and normal subjects (4.7 ± 0.3 ppb, p < 0.05).

### Effect of budesonide and salbutamol inhalation

#### Bronchial blood flow

**Figure 4:**
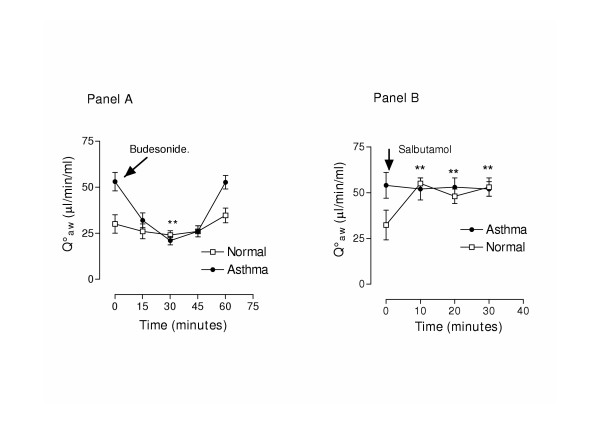
Acute effect of budesonide inhalation (400 μg) (Panel A) and salbutamol (200 μg) (Panel B) on bronchial blood flow (Q_aw_).

In asthmatic patients Q_aw _was significantly reduced 30 minutes after the inhalation of budesonide compared to baseline (53.0 ± 5.0 μl/ml/min and 21.3 ± 2.32 μl/ml/min respectively, p < 0.05 Figure 4 Panel A) and returned to baseline levels at 60 minutes (52.6 ± 4.0 μl/ml/min). In normal subjects there was a tendency for lower Q_aw _after the inhalation of budesonide but such changes were not significant (30.3 ± 5.0 μl/ml/min and 26.3 ± 3.0 μl/ml/min at baseline and at 30 minutes respectively, p > 0.05, n = 5, Figure 4 Panel A).

In 8 normal volunteers Q_aw _was increased after salbutamol inhalation (32.3 ± 8.1 μl/ml/min and 55.0 ± 3.0 μl/ml/min at baseline and at 10 minutes respectively, p < 0.05), while this effect was not present in asthmatic patients (54.0 ± 7.0 μl/ml/min and 52.0 ± 6.0 μl/ml/min, p > 0.05, n = 5, Figure 4 Panel B).

Placebo had no effect on Q_aw _in subjects with asthma (54.0 ± 3.0, 56.1 ± 4.5 and 53.1 ± 8.1 μl/min/ml/ at baseline, 30 and 60 minutes respectively, p > 0.05) nor in normal subjects (34.0 ± 3.0,38.1 ± 4.5 and 35.1 ± 8.1 μl/min/ ml/ p > 0.05)

#### Exhaled breath temperature

**Figure 5:**
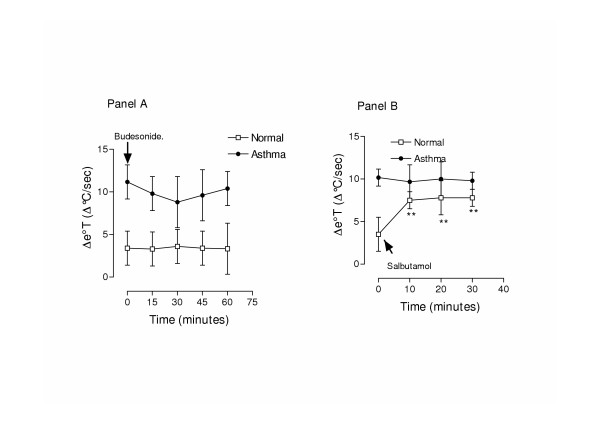
Acute effect of budesonide inhalation (400 μg) (Panel A) and salbutamol (200 μg) (Panel B) on exhaled breath temperature gradients (Δe°T).

The inhalation of budesonide was associated with a tendency for a decrease of Δe°T in asthmatic patients (from 10.17 ± 2 Δ°C/ sec at baseline to 8.6 ± 3 Δ°C/sec 30 minutes after inhalation Figure 5 Panel A), even though this decrease was not significant; the changes in Q_aw _and Δe°T were correlated in asthmatic patients (r = 0.78, p < 0.05). In normal subjects, no significant changes of Δe°T were found at any of the time points after budesonide inhalation (3.5 ± 2 Δ°C/sc at baseline, 3.5 ± 1 Δ°C/sc at 30 minutes, 3.53 ± 2 Δ°C/sc at 60 minutes, p > 0.05).

In 5 normal volunteers Δe°T was increased after the inhalation of 200 μg of salbutamol (3.50 ± 0.29 Δ°C/ sec and 7.8 ± 0.6 Δ°C/ sec, p < 0.01), while this effect was not present in asthmatic patients (10.1 ± 0.44 Δ°C/ sec and 9.67 ± 0.51 Δ°C/ sec, p > 0.05, n = 5, Figure 5 Panel B)

Δe°T was unchanged in subjects with asthma (9.17 ± 2 and 10.23 ± 3.5 Δ°C/ sec at baseline and 10 minutes respectively, p > 0.05) and in normal subjects (3.50 ± 0.29 Δ°C/ sec and 4.20 ± 0.32 Δ°C/ sec p > 0.05) after the inhalation of placebo.

## Discussion

This is the first study to show that elevated levels of Q_aw _and Δe°T are correlated with one another and with airway inflammation as assessed by exhaled NO. We propose that high levels of NO generated in asthmatic patients as a result of airway inflammation may cause vasodilatation of the bronchial circulation contributing to increased heat exchange. This is supported by the demonstration that lower Δe°T levels, after the inhalation of corticosteroids, are correlated with reduced levels of bronchial blood flow. We propose that these non-invasive measurements may be useful to evaluate airway inflammation and may provide a tool to assess steroid sensitivity.

Angiogenesis and microvascular remodelling are features of chronic inflammatory diseases, such as asthma [21]. As inflammatory diseases evolve, the microvasculature undergoes progressive changes in structure and function. Blood vessels enlarge and proliferate supplying inflammatory cells in chronically inflamed tissues. Because of these changes, asthmatic patients have increased vascularity of the airway mucosa which is related to the severity of the disease [2]. Airway vascular remodelling and inflammation maybe responsible for increased bronchial blood flow [22] and exhaled breath temperature gradients in asthmatic patients [5].

In a previous study [23] we have proved that patients with asthma have elevated Δe°T compared to normal subjects and because we found a significant correlation with exhaled NO we suggested that this was due to airway inflammation. In the present study we hypothesised that Δe°T is elevated in asthma as a result of increased bronchial blood flow and we studied the relationship between Q_aw _and Δe°T and airway inflammation as assessed by exhaled NO. Even though the patients enrolled in this study were significantly older than the control group, our previous studies [5,24] indicate that that age does not affect Δe°T. We studied airway inflammation measuring exhaled NO and we investigates its relationship with Q_aw _which has also been suggested as a marker of inflammation. We also studied the interaction of these two parameters after inhaled corticosteroids β_2 _agonists.

We confirmed previous data showing elevated levels of Q_aw _in asthmatic subjects compared to normal volunteers, using a modified method developed by Onorato *et al *[25]. In the current study we preferred the use of acetylene over dimethyl ether because the latter is highly explosive when in contact with oxygen. Gas exchange efficiency is largely dependent on solubility, because these two gases have similar physicochemical characteristics we assume that they can be used interchangeably. Furthermore, the measurement of Q_aw _in this study presented the same response pattern and timing to the inahaltion of steroids [12] and beta agonists [26] as previously showed using the dimethyl ether method confirming that the method presented in our study is an acceptable measurement of Q_aw_.

The method used in this study for the measurement of Q_aw _produces results which include the contribution of the dead space and trachea blood flow to the total bronchial blood flow. We acknowledge that the trachea may not be the main site of inflammation in asthma, but inflammation in asthma extends from the larynx to the terminal bronchioles and the tracheal mucosa certainly appears inflamed in many asthmatic patients. When tracheal inflammation occurs there will be a good separation between normal subjects and patients with asthma because the measurements of bronchial blood flow and exhaled breath temperature will particularly reflect the contribution of this part of the respiratory tract

For the first time we have shown, using non-invasive methods, that changes in bronchial blood flow can alter exhaled breath temperature indicating that the bronchial circulation may control airstream temperature. Furthermore, in this study not only we have shown a correlation between Q_aw _and Δe°T but we have also shown that these measurements respond similarly to steroids and beta 2 agonists. Therefore, patients with asthma have a significantly faster rise of breath temperature and Q_aw _and these are correlated. We presume that this is due to the increased vascularity of the bronchial vessels [27] and elevated blood supply and therefore heat transfer across the bronchial wall. Hyperaemia and hyperperfusion are consistent features of tissue inflammation, therefore, the finding of increased exhaled air temperature and bronchial blood flow in asthmatic patients may be due to the elevated levels of exhaled NO which is a marker of inflammation and a potent bronchial vasodilator. Even though the correlation between Q_aw _and Δe°T may appear to be weak, it is noteworthy that the acute changes of these variable was significantly correlated, reinforcing the hypothesis that elevated breath temperature gradients in asthmatic patients may reflect increased bronchial blood flows.

We were able to show a positive correlation between exhaled NO and Δe°T. NO is a gas produced by several types of pulmonary cells, including inflammatory, endothelial and airway epithelial cells. Elevated levels of exhaled NO in asthma [6], and interstitial lung disease [28] are likely to be due to the activation of the inducible form of NO synthase (iNOS) and therefore may reflect airway inflammation, alternatively NO maybe produced by the bronchial epithelium. In addition the activity of iNOS, the inducible enzyme responsible for the synthesis of NO, is temperature-dependent [29], therefore elevated airway temperatures in patients with asthma [5] may induce further synthesis of NO. NO is a potent vasodilator and may play a role in the regulation of bronchial vasomotor tone [10], so that elevated levels of NO may lead to vasodilatation and increased bronchial blood flow as shown by the correlation between exhaled NO and Δe°T. Unfortunately, in the current study, we were unable to establish the contribution of NO produced by the bronchial vasculature compared to the pulmonary circulation.

In this cross-sectional study we could not show any differences of Δe°T or Q_aw _in corticosteroid-treated compared to untreated asthmatic patients, despite the efficacy of steroids in reducing bronchial blood flow [12]. This is consistent with previous studies published by our group and others showing that steroid-treated asthmatic patients have similar Δe°T [5] and Q_aw _[22] compared to untreated patients. One hypothesis is that the vasoconstrictive action of inhaled corticosteroids may have been balanced by β_2 _induced vasodilatation resulting in minimal changes in bronchial artery diameter and blood flow and therefore no changes of Q_aw _and Δe°T. However, these results must be confirmed by placebo controlled studies.

In contrast to the effect of chronic treatment with corticosteroids, the acute inhalation of budesonide caused a significant temporary reduction of Q_aw _which returned to baseline one hour after inhalation. This is also consistent with a previous publication [22], notably, in the current study Q_aw _and Δe°T and their interaction were studied simultaneously in the same group of patients for the first time. Corticosteroids may cause vasoconstriction by numerous mechanisms. They can potentiate the vasoconstrictor actions of noradrenalin and angiotensin II by upregulating their vascular receptors [30]. Furthermore, corticosteroids may inhibit the synthesis of NO [31], thus causing vasoconstriction. In addition to these mechanisms of action, corticosteroids may also have a very rapid action (less than 5 minutes) by inhibiting noradrenalin uptake in bronchial blood vessels [32]. The glucocorticoid-induced vasoconstriction in asthmatics seems to be accompanied by a greater α_1_-adrenergic vasoconstrictor response [26]. This adds further support to a α_1_-adrenergic steroid interplay in the regulation of vascular tone.

Further studies are required, investigating the dose response relationship for inhaled steroids would provide valuable information.

The cardinal signs of inflammation are rubor (redness), calor (heat), tumor (swelling), dolor (pain), and impaired function (functio laesa). Exhaled breath temperature and bronchial blood flow may reflect rubor and calor in the airways and therefore may be markers of tissue inflammation and remodelling as confirmed by the positive correlation between Δe°T, Q_aw _and exhaled NO which was shown for the first time in this study. Measurement of exhaled breath temperature and bronchial blood flow may provide means of detecting airway inflammation and vascular remodelling in a non-invasive way.
